# Acute Stroke Severity Assessment: The Impact of Lesion Size and Functional Connectivity

**DOI:** 10.3390/brainsci15070735

**Published:** 2025-07-09

**Authors:** Karolin Weigel, Christian Gaser, Stefan Brodoehl, Franziska Wagner, Elisabeth Jochmann, Daniel Güllmar, Thomas E. Mayer, Carsten M. Klingner

**Affiliations:** 1 Department of Neurology, Jena University Hospital, Am Klinikum 1, 07747 Jena, Germany; 2 Biomagnetic Center, Jena University Hospital, Am Klinikum 1, 07747 Jena, Germany; 3 Department of Psychiatry and Psychotherapy, Jena University Hospital, Am Klinikum 1, 07743 Jena, Germany; 4 German Center for Mental Health (DZPG), Department of Psychiatry and Psychotherapy, Jena University Hospital, Philosophenweg 3, 07743 Jena, Germany; 5 Medical Physics Group, Institute of Diagnostic and Interventional Radiology, Jena University Hospital, Philosophenweg 3, 07743 Jena, Germany; 6 Section Neuroradiology, Institute of Diagnostic and Interventional Radiology, Jena University Hospital, Am Klinikum 1, 07743 Jena, Germany

**Keywords:** acute ischemic stroke, lesion size, functional connectivity, NIHSS score, rs-fMRI

## Abstract

Background/Objectives: Early and accurate prediction of stroke severity is crucial for optimizing guided therapeutic decisions and improving outcomes. This study investigates the predictive value of lesion size and functional connectivity for neurological deficits, assessed by the National Institutes of Health Stroke Scale (NIHSS score), in patients with acute or subacute subcortical ischemic stroke. Methods: Forty-four patients (mean age: 68.11 years, 23 male, and admission NIHSS score 4.30 points) underwent high-resolution anatomical and resting-state functional Magnetic Resonance Imaging (rs-fMRI) within seven days of stroke onset. Lesion size was volumetrically quantified, while functional connectivity within the motor, default mode, and frontoparietal networks was analyzed using seed-based correlation methods. Multiple linear regression and cross-validation were applied to develop predictive models for stroke severity. Results: Our results showed that lesion size explained 48% of the variance in NIHSS scores (R^2^ = 0.48, cross-validated R^2^ = 0.49). Functional connectivity metrics alone were less predictive but enhanced model performance when combined with lesion size (achieving an R^2^ = 0.71, cross-validated R^2^ = 0.73). Additionally, left hemisphere connectivity features were particularly informative, as models based on left-hemispheric connectivity outperformed those using right-hemispheric or bilateral predictors. This suggests that the inclusion of contralateral hemisphere data did not enhance, and in some configurations, slightly reduced, model performance—potentially due to lateralized functional organization and lesion distribution in our cohort. Conclusions: The findings highlight lesion size as a reliable early marker of stroke severity and underscore the complementary value of functional connectivity analysis. Integrating rs-fMRI into clinical stroke imaging protocols offers a potential approach for refining prognostic models. Future research efforts should prioritize establishing this approach in larger cohorts and analyzing additional biomarkers to improve predictive models, advancing personalized therapeutic strategies for stroke management.

## 1. Introduction

Around 12 million humans worldwide are diagnosed with stroke every year, with a global prevalence of around 100 million people affected [1]. Current estimations predict a further rise in stroke prevalence of 27% by 2047 due to the aging society and increased survival ratio despite better stroke prevention [2]. Therefore, stroke-associated mortality and morbidity remain high in spite of improved treatment options [3]. The risk–benefit assessment of therapeutic interventions in the acute phase is particularly challenging. Especially intravenous thrombolysis and mechanical thrombectomy show great promise but require careful evaluation [4]. Enhancing therapeutic efficacy necessitates a deeper understanding of the complex neural interactions underlying sensorimotor functions, which advanced neuroimaging techniques can improve. All therapeutic interventions aim to salvage or preserve brain tissue, achieving corresponding improvements in functional outcomes. In this context, it is crucial to derive prognostic information regarding expected clinical deficits from structural lesions. An ischemic stroke manifests as localized damage, but the clinical presentation often encompasses a broader array of symptoms. These deficits may result not only from the ischemic lesion itself but also from indirectly affected areas.

The analysis of neuroimaging data plays a crucial role in assessing both the localized and secondary indirect effects of post-stroke conditions and in developing predictive models for patient outcomes. Previous work has focused on various parameters obtained from different types of data [5,6]. In this context, lesion size has been suggested as a prognostic indicator, admittedly with limitations, such as moderate correlation results or reflections on contemporary relevance [7,8]. Particularly through diffusion-weighted imaging (DWI)-based inference regarding both lesion size and patient outcome, significant insights have been gained [9,10,11]. Lesion size is also an essential reference for evaluating the predictive value of innovative parameters such as corticospinal lesion load [12]. In addition to its use as a reference metric, structural and functional disconnection in systematic evaluations show considerable potential [13]. Alongside prognostic factors from neuroimaging, blood-based and cerebrospinal fluid biomarkers can provide additional information for predicting outcomes in ischemic stroke [14,15]. However, despite all endeavors, previous analyses utilizing diverse data types to create outcome prediction models have not reached a clinically applicable level of accuracy.

Moreover, network connectivity analysis has become established in this field. Besides analyzing structural connectivity, which describes anatomical connections between brain areas, investigating functional connectivity is particularly interesting. For data acquisition, resting-state functional Magnetic Resonance Imaging (rs-fMRI) and diffusion tensor imaging (DTI) are not components of routine acute stroke diagnostics. Both can be applied in the acute or chronic stroke phase. Rs-fMRI provides a way to examine the brain’s natural activity by tracking variations in the Blood Oxygenation Level Dependent (BOLD) signal. This technique helps to identify various brain networks, known as Resting State Networks (RSNs). The first rs-fMRI network analysis demonstrated a high functional correlation between the motor cortex of the left and right hemispheres [16,17]. Further stroke studies concentrating on motor deficits have reported a modified intra- and interhemispheric connectivity between motor areas [18,19,20,21,22].

In recent years, machine learning (ML) techniques have gained substantial traction in stroke imaging research, enabling the development of predictive models from high-dimensional Magnetic Resonance Imaging (MRI) data. Approaches such as lesion-symptom mapping, diffusion-weighted imaging (DWI) analysis, and resting-state functional MRI (rs-fMRI) have been analyzed using both classical and advanced ML algorithms [23,24,25,26]. Functional MRI is widely used due to its high spatial resolution, making it a preferred method for localizing brain activity and identifying network-level dysfunctions. Support vector machines, random forests, and convolutional neural networks have demonstrated their potential in modeling complex relationships between imaging features and clinical outcomes [27,28]. At the same time, regression-based methods remain essential tools, particularly in settings with moderate sample sizes. Techniques such as multiple linear regression, Least Absolute Shrinkage and Selection Operator (LASSO) regression, and ridge regression offer high interpretability and allow for the systematic assessment of predictor importance while controlling for overfitting through cross-validation [29,30]. These methods have been successfully applied to functional and structural imaging data, forming a robust framework for the construction and validation of prognostic models. Our study builds upon this foundation by applying multiple linear regression and cross-validation to evaluate the predictive contribution of lesion size and network-level connectivity metrics in acute stroke.

In the acute stroke setting, a variety of neuroimaging and neurophysiological tools—such as electroencephalography (EEG), evoked potentials (EPs), and ultrasound dopplerography (USDG)—are employed to assess brain structure and function [31,32,33]. FMRI, in combination with structural MRI, offers high spatial resolution, enabling the precise localization of lesions and the assessment of functional connectivity changes. However, its temporal resolution is limited by the hemodynamic response, which evolves over several seconds. In contrast, electrophysiological methods such as EEG and EPs provide millisecond-level temporal resolution and are routinely used in intensive care units for the real-time monitoring of brain function [31,32]. Transcranial USDG offers another complementary approach by assessing cerebral blood flow dynamics [33]. While these modalities are well-suited for continuous bedside monitoring, they lack the spatial specificity and whole-brain coverage provided by MRI and fMRI. In this study, we focus on the combined use of structural and functional MRI to examine how lesion size and connectivity alterations contribute to acute stroke severity.

Nevertheless, most research has focused on examining functional connectivity in the long-term progression following stroke, leading to a lack of studies in the acute setting [34,35]. We compare this research project’s neuroimaging parameters and clinical data during the acute stroke phase among the predominant reports investigating functional connectivity in the chronic phase post-stroke. In this regard, our objective was to analyze whether existing parameters such as lesion size and functional connectivity could serve as early markers of stroke severity. Another purpose was to investigate whether incorporating information on functional connectivity improves the accuracy of predicting stroke severity in the acute stage. Our primary hypothesis suggests that stroke-related reductions in sensorimotor RSN connectivity correlate more strongly with patients’ neurological deficits, as measured by the National Institutes of Health Stroke Scale (NIHSS score), compared to the traditional parameter of lesion size. To test this hypothesis, we analyzed the anatomical and functional MRI data of a cohort of 44 patients with acute or subacute subcortical ischemic stroke.

## 2. Materials and Methods

Utilizing various imaging modalities, this study analyzed clinical and imaging data from stroke patients. Standard preprocessing was used to ensure consistency across the data. Key variables, including clinical scores, lesion metrics, and functional connectivity, were measured and statistically analyzed to determine their correlations and predictive values.

### 2.1. Patient Cohort

Forty-four stroke patients (aged 47 to 86 years, mean age 68.11 years; 23 male; and 19 right and 25 left hemispheric strokes) were retrospectively enrolled in this study. All stroke patients were treated acutely in the Department of Neurology, University Hospital Jena, Germany. Upon presentation to the Emergency Department and the acute management of stroke, patients were admitted to our certified stroke unit or neurologically managed intensive care unit. They subsequently received interdisciplinary stroke unit care for a minimum duration of 24 h and were treated according to standardized clinical guidelines [36]. The stroke patients were recruited from the stroke unit and intensive care unit of the University Hospital Jena. A consistent and standardized examination protocol with comprehensive clinical evaluations and medical history reviews was applied to all patients.

The study established the following inclusion criteria for patient selection: (a) acute or subacute single subcortical ischemic stroke located in the anterior, media, or posterior cerebral territories, confirmed by cranial MRI conducted within seven days after stroke and as part of routine clinical practice, (b) no pre-infarction in the same cerebral area, (c) focal neurological symptoms attributable to the ischemic stroke, which include motoric and/or sensory deficits, (d) baseline NIHSS score at the time of admission below 15 points to ensure the inclusion of patients with mild to moderate stroke severity, (e) age of 18 years or older, and (f) no indication of any other neurological diseases that could account for the observed symptoms, assuring that the deficits are solely due to the ischemic stroke. These inclusion criteria were used to create a homogeneous study population to enable a standardized analysis and the basic applicability of the approach presented.

The patients had experienced mild or moderate strokes, as determined by an NIHSS score of less than 15 points. Specifically, mild strokes were categorized by an NIHSS score ranging from 0 to 6 points, while moderate strokes were indicated by scores ranging from 7 to 14 points. Including patients with an NIHSS score under 15 points at admission was a strategic decision based on several considerations. Firstly, we targeted patients with mild to moderate stroke severity to ensure feasibility for practical participation. Severely affected patients often experience complicated clinical courses, including prolonged intensive care stays, which could hinder consistent study participation. Secondly, this selection criterion aimed to create a more homogeneous cohort, reducing variability caused by extreme stroke severity and allowing a more explicit analysis of functional impacts.

Alongside gathering sociodemographic data, we meticulously collected a range of parameters and clinical information. This included long-term medication regimens, such as (dual) platelet aggregation inhibition and anticoagulation therapy. Acute therapeutic measures were also documented, encompassing systemic thrombolysis, mechanical thrombectomy, and acute thrombendarteriectomy of the carotid artery. Furthermore, the duration of hospitalization was systematically recorded to provide an overview of each patient’s clinical course.

Exclusion criteria were acute visual disturbances (such as hemianopsia), pre-existing motor or sensory deficits resulting from a prior stroke in the same cerebral area, and contraindications for MRI examination, like the presence of non-MRI-compatible implants or incompatibility with gadolinium-containing contrast media.

The patient cohort’s comprehensive demographic and clinical features are delineated in Table 1.

All patients underwent cerebral high-resolution 3 Tesla MRI, which revealed imaging evidence of acute or subacute ischemic stroke. In detail, the anatomical and functional MRI of the patients were obtained, including T1- and T2-weighted imaging data and conventional rs-fMRI. Morphological imaging findings (affected brain side and location of ischemic lesions) were collected from the datasets.

To provide a detailed overview of the lesion distribution and stroke etiology within the cohort, Table 2 summarizes the affected anatomical regions as well as the classification of stroke subtypes. The categorization was made according to the criteria of the classification system from the Trial of Org 10172 in Acute Stroke Treatment (TOAST): (1) large artery atherosclerosis, (2) cardioembolism, (3) small vessel occlusion, (4) stroke of other determined etiology, or (5) stroke of undetermined etiology [37]. Ischemic lesions most commonly involved the basal ganglia, followed by the thalamus and periventricular white matter. Etiologically, strokes were predominantly cardioembolic or cryptogenic, with smaller proportions attributed to microangiopathic and macroangiopathic origins.

In total, we analyzed data collected between December 2019 and November 2021.

The Ethics Committee of Friedrich Schiller University Jena reviewed and approved the study, ensuring compliance with the principles of the Declaration of Helsinki.

### 2.2. Clinical Appraisal

The stroke patients received a neurological examination including the NIHSS score and the modified Rankin Scale (mRS). Both evaluation procedures were documented in the acute phase as well as the follow-up. Neurological impairments were assessed and quantified using the NIHSS score, a standardized scoring system generally considered appropriate for assessing stroke severity [38]. This score can be used effectively for the initial assessment and longitudinal monitoring of stroke-related deficits. Patients’ NIHSS scores were systematically collected by trained neurologists, including residents and specialists, to ensure consistency and accuracy in the assessment. The mRS, currently used extensively in its widely accepted modification, is a measure for assessing the level of disability post-stroke [39]. It was used to evaluate the overall degree of impairment due to stroke and represents a more general assessment, possibly influenced by other factors, including clinical and demographic variables.

### 2.3. MRI Acquisition

All patients in the research project underwent cerebral MRI scans including anatomical and functional MRI data. The MRI examinations were performed using a 3 Tesla MRI scanner (Skyra, SIEMENS, Erlangen, Germany), conducted within a window of seven days following the patients’ admission to the hospital, with the mean interval being 2.48 days (±1.81 SD, median 2.00, range: 0–6). A standardized MRI protocol was uniformly applied across all subjects, ensuring consistency and reliability of the imaging data. This imaging protocol incorporated a high-resolution T1-weighted dataset with an isotropic voxel size of 1 mm (millimeter) and a T2-weighted fluid-attenuated inversion recovery (FLAIR) sequence [40] that maintained identical spatial resolution. The T1-weighted anatomical images were obtained using a three-dimensional (3D) magnetization-prepared rapid acquisition gradient-echo (MPRAGE) sequence [41], with the following settings: voxel size 1 mm isotropic, number of sagittal slices: 176, slice thickness 1 mm, repetition time (TR) 2300 milliseconds (ms), echo time (TE) 306 ms, and inversion time (TI) 1100 ms. Analogously, the FLAIR sequence was acquired using the following parameters: voxel size 1 mm isotropic, number of sagittal slices: 176, slice thickness 1 mm, TR = 5000 ms, TE = 394 ms, and TI = 1800 ms. Alongside anatomical MRI datasets, all patients received fMRI scans using an echo-planar imaging (EPI) sequence during resting-state (task-free period) with the following settings: number of images: 260, slice thickness 2.3 mm, TR 1780 ms, TE 30 ms, flip angle (FA) 90, and percent phase field of view (FOV) 100. The rs-fMRI sequence used in our study had a pure acquisition time of 7.57 min. During the scanning process, the patients were instructed to remain motionless with their eyes closed, while staying alert. They were supposed to avoid focusing on any specific thoughts. To prevent motion artifacts, several preventive measures were applied, including physical stabilization of the head using foam cushions and head fixation devices, complemented by patient instruction and calming measures.

### 2.4. Image Processing and Data Evaluation

**Figure 1:**
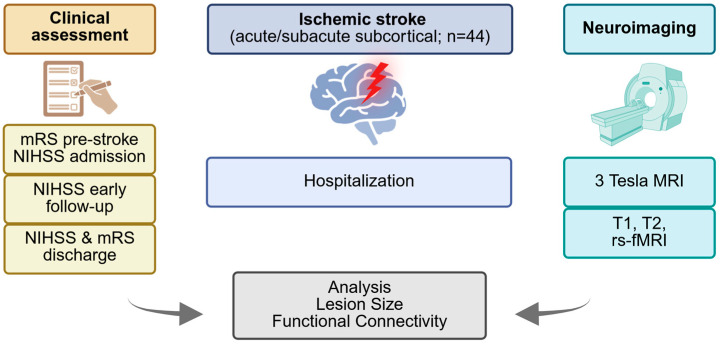
Schematic overview of the analysis framework. The procedure includes acquiring clinical and imaging data of stroke patients, processing anatomical and functional images to determine lesion size and functional connectivity, and investigating the predictive value of these parameters for the clinical status after stroke.

The initial raw datasets comprised structural and functional MRI data. Upon acquisition, all MRI images underwent a meticulous and comprehensive preprocessing pipeline. Figure 1 provides a general insight into the procedure.

#### 2.4.1. Structural MRI Preprocessing

The preprocessing was executed using neuroimaging software tools: Statistical Parametric Mapping (SPM12) [42] and the Computational Anatomy Toolbox (CAT12) [43]. Integrating these tools for preprocessing facilitated the accurate detection and analysis of ischemic lesions within the FLAIR images. The detailed preprocessing process ensured the datasets were precisely prepared, allowing for the high-resolution and high-fidelity analysis of anatomical and pathological features presented in the MRI images. By utilizing SPM12 and CAT12, the study leveraged advanced image processing techniques to handle the complexity of MRI data, ensuring that the resulting datasets were optimized for subsequent statistical analyses and interpretations.

To ensure data quality and consistency, all T1-weighted images were visually inspected for motion artifacts and successful brain extraction. We utilized in-house software under expert supervision to generate lesion masks from stroke patient images, combining manual and automated approaches to ensure accuracy. Initially, MR images were labeled according to their respective modalities and reoriented from sagittal to axial views. To enable consistent normalization, the anterior commissure was marked, and in cases of misalignment—particularly with oblique acquisitions—additional alignment steps were performed. The axis along the falx cerebri was manually adjusted where necessary. Acute ischemic lesions were then comprehensively identified and delineated on each FLAIR slice through expert visual inspection. To support this process, automated lesion segmentation was performed using the Lesion Segmentation Tool (LST) [44], which also enabled lesion filling to improve normalization. Lesion masks and anatomical images were subsequently co-registered using normalized mutual information in SPM12 and transformation into the Montreal Neurological Institute (MNI)-defined standard space was achieved via CAT12. All lesion masks underwent quality control by at least two trained raters at multiple processing stages, with final lesion boundaries confirmed through comparison to the native space and normalized anatomical images.

Following the creation of lesion masks, FLAIR images were co-registered to the T1-weighted image using normalized mutual information in SPM12, and registration parameters were applied to the lesion masks. The LST was used to segment stroke lesions accurately, and the lesions were filled in the T1 image to mitigate biases in spatial registration. This filled T1 image was transformed to the standardized MNI152NLin2009cAsym space using CAT12, and the transformation was also applied to the co-registered stroke lesion mask.

The lesion size was quantified using volumetric calculation functions. The number of voxels comprising the lesion was determined and multiplied by the voxel size (isotropic voxel size of 1 mm). The calculated volume was expressed in cubic millimeters and exported for subsequent analysis.

#### 2.4.2. Preprocessing of rs-fMRI

The preprocessing of rs-fMRI data was performed using MATLAB 9.8.0.1323502 (R2020a) and SPM12. Raw data were assessed for artifacts and excessive head motion (defined as >3 mm translation or >3° rotation), and subjects exceeding these thresholds were excluded. Initially, motion correction was applied to compensate for displacements arising from unavoidable movements during image acquisition. This was achieved by realigning all images to the first image of the series, ensuring consistent spatial orientation. Subsequently, the slice timing correction was applied. The process involved interpolating the acquisition times of individual slices to a uniform time point. The fMRI data were non-linearly co-registered to achieve higher spatial accuracy with the corresponding high-resolution T1-weighted anatomical images. These T1-weighted images had previously been segmented into cerebrospinal fluid (CSF), white matter (WM), and gray matter (GM). Normalization was then performed to transform the data into the standard space defined by the Montreal Neurological Institute. Temporal filtering was applied to enhance signal quality and reduce the impact of non-neuronal noise. Data were filtered between 0.01 and 0.1 Hz to minimize the influence of physiological noise and to focus on low-frequency fluctuations indicative of functional connectivity. This frequency range is widely accepted and has demonstrated robustness in both healthy individuals and patients with stroke across multiple studies. Although no cohort-specific optimization of the frequency band was performed, this standard range has been shown to reliably capture functionally relevant BOLD signal fluctuations in similar clinical contexts. Further studies may evaluate whether tailoring the frequency window could provide added value in acute stroke populations. Finally, spatial smoothing was conducted using a 6 mm full-width at half-maximum (FWHM) Gaussian kernel.

#### 2.4.3. Functional Connectivity Network Analysis

Functional connectivity was analyzed using a seed-based correlation approach, a well-established technique widely employed in numerous studies [17,45]. This method involves selecting regions of interest (ROIs) and examining the correlation between the average BOLD time series of voxels within each ROI and all other brain voxels. ROIs were selected within the atlas based on a hypothesis-driven approach informed by anatomical and functional considerations. This selection was guided by the prior literature on stroke and reflects regions consistently identified as functionally relevant in task-based fMRI studies of stroke patients, although no task-based data were available in the current cohort for direct validation.

For further analysis, we focused on the motor network, the default mode network (DMN), and the frontoparietal network. The anatomical delineation of regions was performed using the AAL3 atlas, which provides the automated anatomical parcellation of a spatially normalized high-resolution T1 volume [46]. This allowed us to map neural networks in greater detail, enhancing our understanding of the functional organization of the selected brain regions. Predefined networks were defined as sets of brain areas involved in specific functions, and functional connectivity was assessed within these networks. The following AAL3 atlas regions were used for network analysis:

- Motor Network I: Precentral Gyrus (PreCG), Postcentral Gyrus (PoCG, contributes to sensorimotor integration), and Paracentral Lobule (PCL).
- Motor Network II: Supplementary Motor Area (SMA), Superior Frontal Gyrus—Dorsolateral (SFG), Middle Frontal Gyrus (MFG), and Inferior Frontal Gyrus—Opercular Part (IFGoperc), Rolandic Operculum (ROL), and Superior Frontal Gyrus—Medial (SFGmedial).
- Motor Network III: Insula (INS), Supracallosal Anterior Cingulate Cortex (ACCsup), and Middle Cingulate Cortex (MCC).
- Motor Network IV: Putamen (PUT), Pallidum (PAL), and Caudate Nucleus (CAU).
- Default Mode Network (DMN): Medial Superior Frontal Gyrus (SFGmedial), Posterior Cingulate Cortex (PCC), Subgenual Anterior Cingulate Cortex (ACCsub), Pregenual Anterior Cingulate Cortex (ACCpre), Supragenual Anterior Cingulate Cortex (ACCsup), Angular Gyrus (ANG), and Precuneus (PCUN).
- Frontoparietal Network: Middle Frontal Gyrus (MFG), Inferior Frontal Gyrus—Opercular Part (IFGoperc), Inferior Frontal Gyrus—Triangular Part (IFGtriang), Angular Gyrus (ANG), and Inferior Parietal Gyrus (IPG).

Each ROI’s average BOLD time series was calculated to obtain its seed time series. Intra-network connectivity was determined by calculating the correlations between each pair of regions within the same network. These r-values were transformed to z-values using Fisher’s Z-transformation, and the z-values were averaged to measure overall intra-network connectivity.

Similarly, inter-network connectivity was estimated by calculating correlations between regions from different networks. These correlations were transformed to z-values and averaged to produce an overall inter-network connectivity measure.

### 2.5. Statistical Analysis

**Figure 2:**
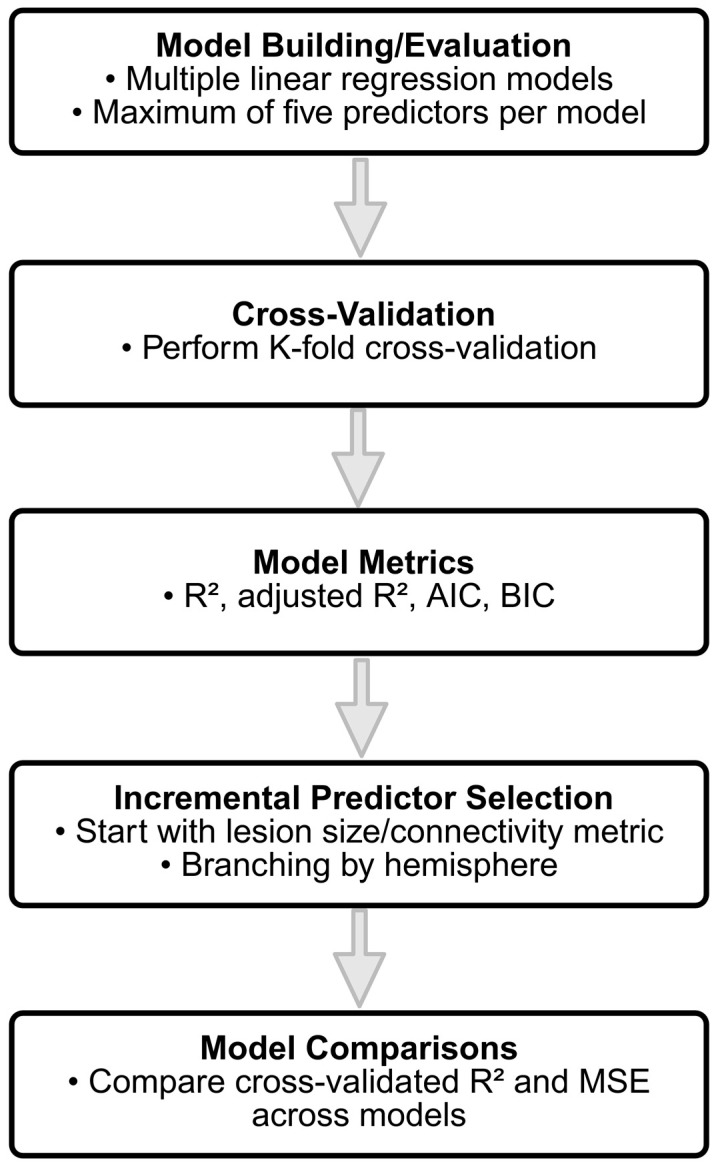
Model selection workflow for identifying the best connectivity predictor. Stepwise comparisons based on lesion size, connectivity metrics, and their combination.

The analysis was performed using multiple linear regression models to assess the relationship between network connectivity metrics and clinical outcomes, measured by NIHSS scores. Statistical modeling was conducted using Python 3.8.3, explicitly leveraging the statsmodels and scikit-learn libraries to evaluate the predictive power of various sets of predictors. Figure 2 illustrates the model selection process used to identify the best connectivity predictor.

The following sections describe the statistical methodology in detail:

- Model Building and Evaluation: To evaluate the predictive capabilities of the different connectivity features, multiple linear regression models were constructed incrementally, with a maximum of five predictors per model to avoid overfitting. The initial model was built using a single predictor, and additional predictors were added based on the increment in R-squared, representing the proportion of the variance explained by the model. Predictors that contributed the most significant increase in R-squared were selected iteratively until the limit of five predictors was reached. The restriction to five predictors was guided by the relatively small sample size (*n* = 44) and the widely recommended rule of thumb requiring a minimum ratio of 10–15 observations per predictor to ensure model stability and generalizability [47]. To ensure validity of the linear regression assumptions, we additionally examined multicollinearity using the Variance Inflation Factor (VIF), ensuring all VIF values remained below two. Normality of residuals was checked visually using Q–Q plots and tested via the Shapiro–Wilk test, confirming approximate normality across models.
- Cross-Validation: To ensure that the model generalizes well to new data, a K-fold cross-validation approach was employed. Specifically, a 5-fold cross-validation was used, where the data was randomly partitioned into 5 equal-sized folds. For each fold, the model was trained on four of the folds and tested on the remaining one. This process was repeated five times, with each fold serving as the test set once, and the results were averaged to obtain the cross-validated R-squared and mean squared error (MSE). The cross-validated R-squared provides an estimate of how well the model is expected to perform on unseen data, mitigating the risks of overfitting.
- Model Metrics: For each model, key statistical metrics were computed, including the R-squared, adjusted R-squared, Akaike Information Criterion (AIC), and Bayesian Information Criterion (BIC). R-squared represents the proportion of variance in the outcome variable explained by the model. Adjusted R-squared accounts for the number of predictors in the model, providing a more conservative estimate compared to R-squared, especially for models with multiple predictors. AIC and BIC are measures of model quality, with penalties for the number of predictors, used to compare models and prevent overfitting.
- Incremental Predictor Selection: To identify the best set of predictors, two incremental model selection approaches were employed: (1) models starting with lesion size as the initial predictor, and (2) models without lesion size, focusing on connectivity metrics. In the incremental approach, the predictor that led to the largest increase in the model R-squared value was added iteratively until a total of five predictors were included. Additionally, models were built separately for different hemispheres, including left hemisphere predictors, right hemisphere predictors, and predictors from both hemispheres, to explore the contributions of region-specific connectivity metrics.
- Model Comparisons: The performance of each model was assessed by comparing cross-validated R-squared and MSE across different models. Cross-validation allowed for an unbiased estimation of model performance on new data, while the use of different subsets of predictors allowed for detailed insights into the relative importance of lesion size versus connectivity-based predictors in explaining clinical outcome. In particular, separate models were built to explore the predictive power of left hemisphere, right hemisphere, and bilateral (both hemispheres) connectivity metrics, providing insights into the specific contributions of different brain regions.

## 3. Results

**Figure 3:**
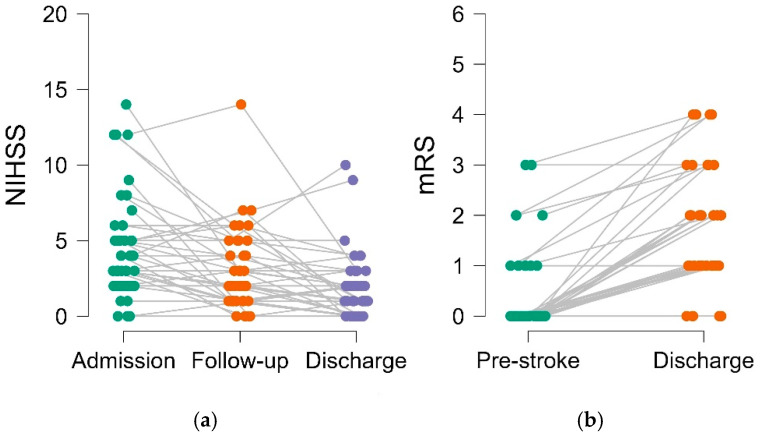
Clinical patient trajectories with paired measurements and assessment intervals in two panels: (**a**) National Institutes of Health Stroke Scale (NIHSS) at admission, early follow-up, and discharge. The observed shift to lower NIHSS values across the time points indicates neurological improvement within the cohort. (**b**) Modified Rankin Scale (mRS) pre-stroke and at discharge. The plot captures the variability between individual patients at the measurement time points.

Our study encompassed 44 patients in total, with 19 experiencing strokes in the right hemisphere and 25 in the left hemisphere. Upon arrival at the Emergency Department, all patients exhibited acute focal neurological symptoms. The average baseline NIHSS score was 4.30 points (±3.35 SD, median 3.00, and range: 0–14), and the NIHSS score 24 h after admission (early follow-up) was 2.86 points (±2.60 SD, median 2.00, and range: 0–14). At the time of discharge, the NIHSS score was 1.84 points (±2.17 SD, median 1.00, range: 0–10, and *n* = 43) and the mRS 1.73 (±1.18 SD, median 1.50, range: 0–4, and *n* = 40). The mean duration of hospitalization was 6.64 days (±2.84 SD, median 6.00, and range: 2–14). The distributions of NIHSS and mRS across the evaluated time points are presented in Figure 3.

Resting-state functional connectivity was assessed for regions defined by the AAL3 atlas, comprising six functional networks. Connectivity estimates were obtained separately for the left hemisphere, right hemisphere, and both hemispheres, resulting in 18 network configurations. In total, 171 connectivity values (predictors) were calculated, including intra-network and inter-network connectivity.

Initially, we tested the predictive power of lesion size versus each individual connectivity predictor. This comparison aimed to determine whether single connectivity metrics provided predictive accuracy comparable to lesion size.

To evaluate the predictive ability of lesion size and connectivity metrics, multiple linear regression models were constructed and compared. A summary of the model metrics for each approach is provided in Table 3, including R-squared, adjusted R-squared, AIC, and BIC. Cross-validation metrics, such as cross-validated R-squared and MSE, were also used to evaluate generalizability.

The model using lesion size only, which was treated as a continuous variable, explained approximately 48% of the variance in NIHSS scores (R-squared = 0.48, cross-validated R-squared = 0.49), indicating a moderate predictive ability. No binarization or stratification of lesion size was applied, as continuous modeling preserves predictive information and avoids arbitrary threshold effects. In contrast, the model using the connectivity metric (bilateral primary motor vs. left basal ganglia motor) only had a lower R-squared value of 0.21, suggesting that lesion size was a more robust single predictor of clinical outcome compared to individual connectivity metrics.

When building incremental models without lesion size, the best set of predictors achieved an R-squared of 0.56, with a cross-validated R-squared of 0.59. Including lesion size as the first predictor resulted in a notably higher R-squared of 0.71 and a cross-validated R-squared of 0.73, demonstrating the added predictive power of lesion size combined with connectivity metrics.

Models built for specific hemispheres provided differential predictive performance. The left hemisphere model showed the strongest fit among hemisphere-specific approaches, achieving an R-squared of 0.54 (adjusted R^2^ = 0.48) and a mean cross-validated R^2^ of 0.57 (folds: 0.559, 0.564, 0.612, 0.572, and 0.543). This outperformed both the right hemisphere model (R^2^ = 0.42, CV R^2^ = 0.45) and the bilateral model (R^2^ = 0.48, CV R^2^ = 0.52). A paired *t*-test across cross-validation folds confirmed a statistically significant improvement for the left hemisphere model compared to the right (*p* = 0.0016), and a smaller but still significant difference compared to the bilateral model (*p* = 0.0454). AIC values further supported this result: ΔAIC for the left vs. right hemisphere model was 10.40, and for the left vs. bilateral model was 5.95, favoring the left hemisphere in both cases. Additionally, a likelihood ratio test showed that extending the primary motor-only model with left-lateralized incremental predictors resulted in a significantly better fit (χ^2^ = 24.42, df = 4, and *p* = 0.0001). These findings indicate that left-lateralized functional connectivity, particularly within and across motor and attention-related networks, is more strongly associated with acute NIHSS scores than right-sided or interhemispheric features. This aligns with the known lateralization of motor control and the dominance of the left hemisphere in skilled movement planning.

Overall, the findings indicate that lesion size is a strong predictor of NIHSS scores, and combining lesion size with carefully selected connectivity metrics yields the best predictive performance. Connectivity metrics alone, while informative, did not achieve the same level of predictive accuracy as when lesion size was included.

**Figure 4:**
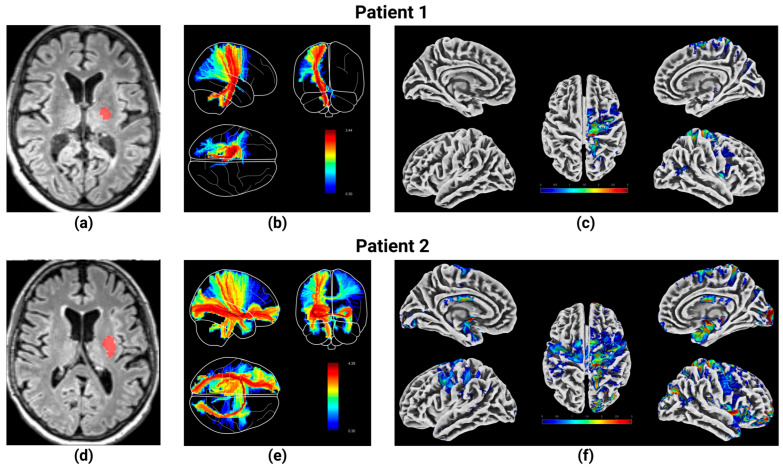
Representative MRI example from two patients with left-hemispheric subcortical ischemic stroke. **Top row**: unilaterally affected patient 1; **bottom row**: bilaterally affected patient 2. (**a**,**d**) Original FLAIR image with the outlined lesion in red. (**b**,**e**) Functional connectivity network originating from the lesion. (**c**,**f**) Cortical regions affected by the lesion and its connectivity.

To further contextualize these results and provide a visual representation of the underlying data, Figure 4 presents representative MRI data from two patients with a left-hemispheric subcortical ischemic lesion. Displayed are (a) anatomical sequences with the lesion highlighted in red, (b) the corresponding connectivity maps, and (c) a multidimensional depiction of the affected cortex. This was implemented exemplarily for one patient with unilateral involvement and another with bilateral involvement. These cases are representative of the cohort and support the observed relationship between lesion size and connectivity.

## 4. Discussion

The study demonstrates that lesion size robustly predicts NIHSS scores in the acute stroke phase. Combining lesion size with strategically selected connectivity metrics enhances predictive performance. Although connectivity metrics provide valuable insights, their predictive value does not match the effectiveness achieved when considering lesion size. Consequently, this underscores the potential of compounding the classical parameter lesion size with functional connectivity to predict the clinical status of acute stroke patients.

Our results add to the comprehensive discussion about the interaction between lesion metrics and functional connectivity with clinical stroke severity. In the clinical setting, determining lesion location and quantifying lesion size enable the primary assessment of the extent of brain damage. A variety of studies identified lesion size as a prognostic indicator of outcomes following acute ischemic stroke, with limitations [48,49,50,51,52]. Nevertheless, the lesion size provides information for clinical decision-making, both for acute interventions such as thrombolysis or thrombectomy and in the context of rehabilitation strategies, for the evaluation of therapies, and for the clinical prognosis to set realistic expectations for recovery. The finding that lesion size alone may not provide a complete explanation for functional impairment caused by stroke is generally recognized and confirmed by our results.

Furthermore, previous research affirmed the predictive relevance of functional connectivity in stroke patients. The analysis of functional neuroimaging data according to stroke has shown changes at several levels and can provide insights into network reorganization processes [53]. In particular, reorganizing the sensorimotor network and corticospinal tract after subcortical stroke is integral in supporting and improving functional recovery [54]. Further results indicate that the functional link between the limbic and dorsal attention networks after a subcortical stroke could serve as a predictor for long-term motor function and neurological deficit outcomes [55]. Previous work has demonstrated a substantial functional correlation between the motor cortices of the left and right hemispheres [17]. Various rs-fMRI studies have also observed reduced interhemispheric connectivity between motor areas [18,19,21,56,57,58]. In this context, functional connectivity between motor areas across the two hemispheres has been identified as a significant determinant of functional outcomes after acute ischemic stroke [34]. Thus, it was concluded that functional recovery following stroke is correlated to the maintenance of the functional connectivity of motor and non-motor networks [59]. Further behavioral deficits after stroke show low dimensionality, reflected in broad yet consistent alterations in functional connectivity, such as reduced modularity [60]. Another study based on over 1000 stroke lesions introduced the human disconnectome, showing that brain disconnections shape functional organization and providing an atlas of white matter function to improve predictions and support cognitive research [61]. Moreover, a novel stroke lesion network mapping approach demonstrated increased specificity of disconnected systems and their correlation with post-stroke behavioral impairments, yet it did not yield improved prediction models for clinical deficits [62]. Salvalaggio et al. had earlier described that the indirect inference of structural disconnection provides predictive value for post-stroke behavioral impairments analogous to lesion-based models, whereas estimates of functional disconnection lacked predictive utility and could not replace direct measures of functional connectivity [63]. Recently, evidence has emerged suggesting that integrating multivariate lesion-behavior mapping with lesion network analysis improves the prediction of long-term post-stroke impairments [64]. All of this work contributes to the expanding body of evidence on brain networks and the application of advanced neuroimaging techniques [65,66,67].

With regard to the asymmetric organization of functional connectivity in the human brain, certain networks tend to exhibit dominance patterns that are influenced by individual differences. In this context, our hemisphere-specific finding that the left hemisphere model yielded higher R-squared values can be viewed in relation to previously described functional asymmetries between the hemispheres. Previous work supports the existence of hemispheric asymmetry, demonstrating higher intrahemispheric connectivity in the left hemisphere—particularly in regions associated with linguistic functions and precise motor control—while spatial perception and attention in the right hemisphere appears to engage in a more integrative manner across both hemispheres [68]. Additionally, sex-dependent hemispheric asymmetries have been reported, with males showing a more pronounced leftward asymmetry [69].

Our study shows the feasibility of performing rs-fMRI in the early stroke phase. The option provides the opportunity to implement early and targeted therapeutic interventions. This is because functional connectivity in fMRI appears to be an imaging biomarker for acute ischemic stroke and a potential treatment target [34]. Neurostimulation techniques such as transcranial magnetic stimulation (TMS) can enhance motor cortex excitability, but its therapeutic efficacy remains debated [70]. Nevertheless, the functional connectivity of motor cortices, assessed using rs-fMRI, can help identify patients at high risk for unfavorable functional outcomes who may benefit from neurostimulation therapies. So, TMS can potentially become an integral component of stroke therapy [71]. Consequently, initiating rehabilitative treatments including neurostimulation techniques early on, right within the acute phase in the stroke unit, could be a future-oriented practice in stroke neurorehabilitation.

While rs-fMRI provides valuable insights due to its high spatial resolution and ability to detect network-level dysfunctions, it is important to acknowledge the alternative modalities described in the introduction (EEG, EPs, and USDG). These techniques offer superior temporal resolution—on the order of milliseconds compared to the several-second delay of hemodynamic responses captured by fMRI—enabling real-time monitoring, which is particularly advantageous for bedside applications. However, their spatial resolution and capacity to assess deep brain structures and network-level alterations are limited compared to fMRI. In contrast, the combined use of rs-fMRI with multiple linear regression and cross-validation, as demonstrated in our study, offers a refined prognostic approach capable of identifying the early markers of stroke severity. Our results, which demonstrate that lesion size alone and in combination with connectivity metrics, explain a substantial proportion of the variance in NIHSS scores and can be compared with the predictive accuracy reported for EEG based approaches [72]. Moreover, EP measures, such as those derived from TMS, may serve as valuable indicators for predicting functional recovery after stroke [73]. While electrophysiology-based approaches show promise, their spatial and integrative capacities are limited compared to MRI-based models, highlighting the potential of multimodal MRI integration for clinical decision support.

An overarching objective of this study is to evaluate early neuroimaging markers for stroke severity and to stratify patients according to their prognosis at an acute stage. The recording of both the clinical and the anatomical MRI data can be performed in the clinical setting without any significant difficulties. Moreover, a benefit is the capability to integrate rs-fMRI into routine clinical MRI protocols. The procedure remains challenging, of course, due to several practical limitations. First, the required scan duration (7.57 min in our study) may exceed patient tolerance in the acute setting and increase susceptibility to motion artifacts. Patient-related factors such as reduced consciousness, agitation, or clinical instability further complicate image acquisition and data quality. Despite these barriers, clinical experience shows that after careful selection stroke patients can tolerate rs-fMRI without serious problems. Second, the availability of advanced MRI infrastructure and trained personnel for data preprocessing and analysis is limited in many clinical settings, particularly in non-tertiary stroke centers. Third, acute stroke workflows are typically optimized for time-critical decisions based on structural imaging (e.g., DWI, computed tomography), which can be completed more rapidly and are essential for treatment selection. As a result, rs-fMRI remains primarily a research tool, with future clinical integration dependent on technical advances in motion correction, automated processing pipelines, and streamlined protocols that minimize acquisition time and complexity. Addressing these barriers is critical for the eventual translation of connectivity-based biomarkers into routine stroke care. As described previously, diverse potential biomarkers that can predict the likelihood of functional recovery after stroke have been identified in existing studies. Future research should consider integrating broader biomarkers from large databases and employing techniques to develop algorithms that more accurately predict patients’ recovery potential post-stroke [74]. In the context of our results, lesion size and functional connectivity can predict the clinical condition of stroke patients in the acute phase.

It is crucial to acknowledge the following limitations of our study: First, our cohort was restricted to subcortical ischemic strokes, yielding relatively homogeneous motor–sensory deficits. Consequently, generalizability to purely cortical stroke populations is uncertain: cortical lesions often span multiple functional networks (e.g., language, attention, and memory), producing more heterogeneous clinical profiles and may compromise BOLD-signal fidelity in cortical ROI seeds. Second, although a whole-brain rs-fMRI analysis would offer broader insight, cortical lesion cohorts require specialized preprocessing—such as cost-function masking [75] or enantiomorphic normalization [76]—and ROI redefinition to mitigate distortion from large cortical infarcts. Third, we did not perform task-based fMRI, which in cortical strokes is particularly valuable for dissociating network dysfunction across cognitive and motor domains. Fourth, our primary outcome was acute NIHSS, which emphasizes motor and language impairments but underrepresents cognitive and high-order deficits common in cortical strokes; future work should incorporate domain-specific scales (e.g., Fugl-Meyer score for sensorimotor impairments, Montreal Cognitive Assessment for cognition). Fifth, symptom and lesion-location heterogeneity in cortical cohorts (e.g., aphasia vs. visuospatial neglect) may further reduce statistical power and necessitate domain-specific subanalyses or larger, stratified samples. The general population of stroke patients is characterized by heterogeneity in several aspects, such as type of stroke, lesion characteristics, initial motor impairment, or vascular risk factors. Furthermore, treatment-related heterogeneity exists, as some patients received thrombolysis and/or thrombectomy. While we documented these interventions, treatment variables were not included in the final models to avoid overfitting, given the limited sample size and our focus on imaging-derived predictors. Nevertheless, we recognize that future studies with larger cohorts and detailed treatment timing may uncover additional variances explained by therapeutic interventions. Sixth, technical constraints—such as a short scan duration, motion artifacts, and a limited signal-to-noise ratio—remain a concern. In addition, cortical-lesion-specific normalization methods (cost-function masking, enantiomorphic) and advanced parcellation tools (Virtual Brain Grafting (VBG) [77], Virtual Brain Transplantation (VBT) [78]) should be prioritized in future studies to improve reproducibility of network models. Generally, in functional connectivity analysis, the selection of brain structures and potential artifacts must be considered, for which Independent Component Analysis (ICA) can serve as a valuable tool [79]. Overall, specific requirements for stroke MRI processing in stroke research have been widely acknowledged, also with diverse normalization methods [80,81]. Finally, to test whether lesion size and functional connectivity retain their predictive value beyond subcortical strokes, mixed-lesion cohorts with stratification by lesion location are essential. Integrating complementary modalities (EEG, magnetoencephalography) and longitudinal outcome measures will further strengthen generalizability across the full spectrum of ischemic stroke.

## 5. Conclusions

In summary, our findings suggest that integrating lesion size and resting-state functional connectivity (rs-fMRI) yields a reliable and informative prediction of clinical status in the acute phase of ischemic stroke, as measured by the NIHSS score. This combined approach enhances predictive accuracy compared to lesion metrics alone and emphasizes the value of network-based information in explaining early neurological impairment.

The prognostic relevance of our approach extends beyond mere classification. By capturing early alterations in brain network integrity, particularly within the sensorimotor system, rs-fMRI offers the opportunity to stratify patients by recovery potential. This may support individualized rehabilitation planning by identifying patients at risk of poor outcomes who could benefit from targeted interventions such as neuromodulation or intensified therapy. Brain networks—especially those showing disrupted interhemispheric or intra-network connectivity—emerge as meaningful targets for therapeutic modulation. A major advantage of our method lies in its clinical applicability: rs-fMRI is task-free, can be performed within a short scan time, and may be seamlessly integrated into existing stroke imaging protocols within hours of symptom onset. Our data demonstrate its feasibility even in the acute-to-subacute stage, reinforcing its potential role in early decision-making. While this study focused on a single time point during the acute to early subacute phases, follow-up examinations—if acquired in future studies—could be analyzed to assess dynamic reorganization processes during recovery. Evaluating time-dependent changes in connectivity metrics over the course of hospitalization or rehabilitation could further enhance prognostic modeling and should be a priority for future research.

Taken together, this work supports the use of multimodal imaging to improve early functional prognosis after stroke. Future prediction models incorporating both anatomical and functional markers, possibly enriched with longitudinal imaging and behavioral data, may advance personalized stroke care and guide rehabilitative strategies more precisely.

## Figures and Tables

**Table 1:** Demographic and clinical features of the patient cohort including National Institutes of Health Stroke Scale (NIHSS) and modified Rankin Scale (mRS). Values are presented as the mean ± standard deviation (SD) and median with range in parentheses.

N	44
Age (in years)	68.11 ± 10.2 (68.50, 47–86)
Sex female/male (in %)	21/23 (47.73/52.27%)
NIHSS score at admission	4.30 ± 3.35 (3.00, 0–14)
NIHSS score at 24 h (early follow-up)	2.86 ± 2.60 (2.00, 0–14)
NIHSS score at discharge	1.84 ± 2.17 (1.00, 0–10, *n* = 43)
mRS pre-stroke level	0.44 ± 0.84 (0.00, 0–3, *n* = 41)
mRS at discharge	1.73 ± 1.18 (1.50, 0–4, *n* = 40)
Affected hemisphere right/left (in %)	19/25 (43.18%/56.82%)
Premedication	
Platelet aggregation inhibition (single or dual; in %)	11 (25.00%)
Oral anticoagulation (in %)	2 (4.55%)
Acute treatment intervention	
Systemic thrombolysis (external or in-house, in %)	10 (22.73%)
Mechanical thrombectomy (in %)	4 (9.10%)
Both (thrombolysis and thrombectomy)	3 (6.82%)
Acute carotid artery thrombendarteriectomy (in %)	0 (0.00%)
Duration of hospitalization in days	6.64 ± 2.84 (6.00, 2–14)

**Table 2:** Anatomical and etiological characteristics of the stroke cohort. (Trial of Org 10172 in Acute Stroke Treatment (TOAST)).

N	44
Anatomical region	
Basal ganglia	28 (63.63%)
Thalamus	7 (15.90%)
Centrum semiovale and periventricular regions	5 (11.37%)
Other (e.g., hand knob, hippocampus)	4 (9.10%)
Etiological subtype (TOAST classification)	
Cardioembolic	14 (31.82%)
Cryptogenic	12 (27.27%)
Microangiopathic	11 (25.00%)
Macroangiopathic	7 (15.91%)

**Table 3:** Systematic overview of the comprehensive summary of model metrics for each approach (Akaike Information Criterion (AIC), Bayesian Information Criterion (BIC), and mean squared error (MSE)).

Model Description	R-Squared	Adjusted R-Squared	AIC	BIC	Cross-Validated R-Squared	Cross-Validated MSE
Lesion Size Only	0.48	0.47	205.39	208.96	0.49	6.62
Best Connectivity Predictor Only (bilateral primary motor vs. left basal ganglia motor)	0.21	0.19	224.17	227.73	0.21	9.45
Best Predictors Without Lesion Size	0.56	0.50	206.33	217.03	0.59	8.24
Best Predictors Including Lesion Size	0.71	0.67	188.00	198.70	0.73	5.37
Best Predictors for Left Hemisphere Only	0.54	0.48	207.74	218.45	0.57	8.28
Best Predictors for Right Hemisphere Only	0.42	0.35	218.14	228.85	0.45	9.87
Best Predictors for Both Hemispheres (D.)	0.48	0.41	213.69	224.40	0.52	10.75

## Data Availability

The data presented in this study are available on request from the corresponding author due to restrictions (privacy, legal/ethical reasons).

## Abbreviations

The following abbreviations are used in this manuscript:

AIC	Akaike Information Criterion
BIC	Bayesian Information Criterion
BOLD	Blood Oxygenation Level Dependent
CAT	Computational Anatomy Toolbox
CSF	Cerebrospinal fluid
DMN	Default Mode Network
DTI	Diffusion tensor imaging
DWI	Diffusion-weighted imaging
EEG	Electroencephalography
EP	Evoked potential
EPI	Echo-planar imaging
FA	Flip angle
FLAIR	Fluid-attenuated inversion recovery
FOV	Field Of View
FWHM	Full-width at half-maximum
GM	Gray matter
ICA	Independent Component Analysis
LASSO	Least Absolute Shrinkage and Selection Operator
LST	Lesion Segmentation Toolbox
MEG	Magnetoencephalography
ML	Machine learning
MM	Millimeter
MNI	Montreal Neurological Institute
MPRAGE	Magnetization-prepared rapid acquisition gradient-echo
MRI	Magnetic Resonance Imaging
MRS	Modified Rankin Scale
MS	Milliseconds
MSE	Mean squared error
NIHSS	National Institutes of Health Stroke Scale
ROI	Region of interest
RS-FMRI	Resting-state functional Magnetic Resonance Imaging
RSN	Resting state networks
SD	Standard Deviation
SPM	Statistical Parametric Mapping
TE	Echo time
TI	Inversion time
TMS	Transcranial Magnetic Stimulation
TOAST	Trial of Org 10172 in Acute Stroke Treatment
TR	Repetition time
USDG	Ultrasound dopplerography
VBG	Virtual Brain Grafting
VBT	Virtual Brain Transplantation
VIF	Variance Inflation Factor
WM	White matter
